# Modified interproximal tunneling technique with customized sub-epithelial connective tissue graft for gingival papilla reconstruction: report of three cases with a cutback incision on the palatal side

**DOI:** 10.1186/s12903-023-03525-7

**Published:** 2023-10-26

**Authors:** Jia-chen Dong, Yue Liao, Meng-jun Sun, Yin Gong, Hui-wen Chen, Zhong-chen Song

**Affiliations:** 1 Department of Periodontology, Shanghai Ninth People’s Hospital, Shanghai Jiao Tong University School of Medicine, 200011 Shanghai, China; 2 College of Stomatology, National Center for Stomatology, Shanghai Jiao Tong University, Shanghai, China; 3 National Clinical Research Center for Oral Diseases, Shanghai Key Laboratory of Stomatology, Shanghai Research Institute of Stomatology, Shanghai, China; 4 Department of Stomatology, the first affiliated hospital of Soochow University, Suzhou, Jiangsu China

**Keywords:** Gingival papilla reconstruction, Modified interproximal tunneling, Connective tissue graft

## Abstract

**Background:**

Gingival papilla defects, which cause an unpleasant appearance and involve the upper anterior teeth, may be triggered by several factors. Several noninvasive and invasive techniques have been proposed for gingival papilla reconstruction. The combination of interproximal tunneling and customized connective tissue grafts (CTGs) has shown promise in papilla augmentation. However, due to the narrowness and limited blood supply of the gingival papilla, the long-term outcomes of these techniques remain unpredictable. Therefore, achieving tension-free coronal advancement of the interdental papilla and proper placement of the CTG is crucial for successful long-term outcomes and could provide widely applicable methods for papilla augmentation.

**Case report:**

In this study, we enrolled three patients with gingival papilla defects in the maxillary anterior teeth. For reconstruction, we proposed a modified interproximal tunneling (MIPT) technique combined with a CTG. A crucial modification based on previous studies involved adding a cutback incision to the base of the palatal vertical incision, resulting in tension-free healing. Additionally, the CTG was sutured upright to further enhance the height of the gingiva papilla. To evaluate the efficacy of the MIPT technique, the clinical parameters—including the Jemt papilla index and the distance from the tip of the papilla to the interproximal contact point—were examined using a periodontal probe (UNC15, Hu-friedy) at baseline and 12 months after surgery. All three patients achieved satisfactory papilla reconstruction 12 months after the surgery. These three cases were used to evaluate the efficacy of the MIPT technique combined with the customized CTG. An average increase in the Jemt papilla score from 1.6 to 2.8 and a reduction in the distance from the papilla tip to the contact point of adjacent teeth from 2 mm to 0.08 mm were observed 12 months after surgery.

**Conclusion:**

The preliminary results confirmed that this technique holds promise for gingival papilla augmentation between tooth/tooth or tooth/implant.

## Introduction

The height of the gingival papilla is affected by various factors, including anatomical factors, inflammation severity, the distance from the interproximal bone crest to the contact point, and previous nonsurgical/surgical therapy [1]. Among these factors, the distance between the crest of bone and the contact point is the most critical. Tarnow et al. found that when the distance mentioned above was ≤ 5 mm, 98% of the gingival papillae between natural teeth were intact; however, this percentage decreased to 56% and 27% when the distance reached 6 mm and ≥ 7 mm, respectively [1, 2]. Periodontal phenotype is another significant factor affecting the integrity of the gingival papilla. The proportion of gingival papilla loss was found to be higher in patients with a thin phenotype than in those with a thick phenotype [3]. In addition, tooth shape, root angulation, periodontal inflammation, and iatrogenic factors also affect gingival papilla integrity [1, 3, 4].

Gingival papilla deficiency is a complex aesthetic and functional problem. According to the Jemt papilla index [5], gingival papilla defects are classified into five categories based on the degree of filling: 0, which indicates that no papilla is present; 1, which indicates that less than half of the papilla height is present; 2, which indicates that half or more of the papilla height is present; 3, which indicates that the papilla fills up the entire proximal space; and 4, which indicates that the papillae are hyperplasic. The loss of gingival papilla height leads to the formation of a black triangle, which reduces the aesthetics of the anterior teeth. In addition, a gingival papilla defect can result in food impaction and plaque accumulation, further damaging periodontal health. Various nonsurgical treatment approaches have been proposed to reconstruct deficient gingival papillae, including hyaluronic acid injection [6, 7], an orthodontic method to align divergent roots [8], and gingival conditioning through the adjustment of the interproximal contact point [9]. Additionally, many microsurgical techniques have been developed by authors such as Beagle [10], Han and Takei [11], and Sculean and Allen [12]. These surgical methods provide some protection to gingival papilla tissue while preparing the gingival flap. However, due to the narrowness and insufficient blood supply in the interproximal space, few surgical methods have achieved excellent postoperative results. Thus, Feuillet et al. proposed the interproximal tunneling (IPT) technique in combination with a customized connective tissue graft (CTG), reporting promising results in terms of papilla gain [13]. The CTG is extensively used in soft tissue reconstruction and has shown positive effects on gingival reconstruction and reduced recurrence of interdental papilla loss [14].

Previous studies have proposed various approaches to separate the base and labial sides of the gingival papilla in full-thickness flaps while preparing the palatal side as a split-thickness flap. According to the IPT technique, the base of the papilla remains attached to the surrounding tissues on both the buccal and palatal sides [15]. Additionally, the tunneling surgical approach, which eliminates the need for releasing incision, enhances vascularity in the surgical area. However, this can lead to difficulties in reducing tension in the palatal gingiva. Furthermore, achieving tension-free suturing with a palatal vertical incision alone during surgery is challenging [16]. Therefore, reducing tension in the palatal gingiva using the IPT technique is difficult, limiting the height of the reconstructed gingival papilla and hindering the insertion of a thicker CTG. Hence, in this study, we describe a modified interproximal tunneling technique (MIPT), which involves a cutback incision on the palatal side and rearrangement of CTG. This modification improves the shape of the palatal incision. We also present a case series evaluating the effects of MIPT with CTG on gingival papilla regeneration between tooth/tooth, pontic/pontic, tooth/pontic, and tooth/implant. The three cases in this study represent most clinical cases requiring gingival papilla reconstruction.

## Case presentation

This study included three females (an average age of 24 years) with gingival papilla defects in the maxillary anterior teeth that seriously affected their facial aesthetics. All the patients satisfied the following inclusion criteria: (I) had no systemic diseases, (II) were not pregnant, (III) did not smoke, and (IV) had a full-mouth plaque score and a full-mouth bleeding score of ≤ 10%. Before the surgery, oral hygiene instruction and periodontal initial therapy were provided to the patients. The three patients’ demographic data and clinical parameters are described in Tables 1 and 2. Also, the gingival biotypes of three patients were evaluated with periodontal probe transparency method [17]. All the patients received periodontal initial therapy and subsequent surgical treatment at the Department of Periodontology, Shanghai Ninth People’s Hospital, Shanghai Jiao Tong University School of Medicine. The study was conducted following the Declaration of Helsinki (as revised in 2013). The study was approved by the institutional board of Shanghai Ninth People’s Hospital, Shanghai Jiao Tong University School of Medicine (Ethical consent NO. SH9H-2021-A711-1), and informed consent was signed by all the patients.

**Table 1:** Demographic data

Characteristic	Patient 1	Patient 2	Patient 3
Age	22	26	24
Sex	Female	Female	Female
BMI	19.5	18.2	20.3
Phenotype	thin	thick	thick
Oral hygiene status	DI: 1 CI: 0	DI: 0 CI: 0	DI: 1 CI: 0
Type of crown form	Compound	Narrow	Square
Type of gingival embrasures	II	II	II

BMI: body mass index

**Table 2:** Baseline and 12-month postoperative values for clinical parameters

Clinical parameter	Baseline	Post-operation	*p* Value
BI	1.83 ± 0.39	1.00 ± 0.85	0.024
PD/mm	1.75 ± 0.44	1.64 ± 0.49	0.048
CAL/mm	1.92 ± 0.51	1.33 ± 0.49	0.282
REC-HT/mm	2.00 ± 0.85	0.08 ± 0.19	0.002

BI: Bleeding index; PD: Probing depth; CAL: Clinical attachment level; REC-HT: Gingival recession height

**Fig. 1:**
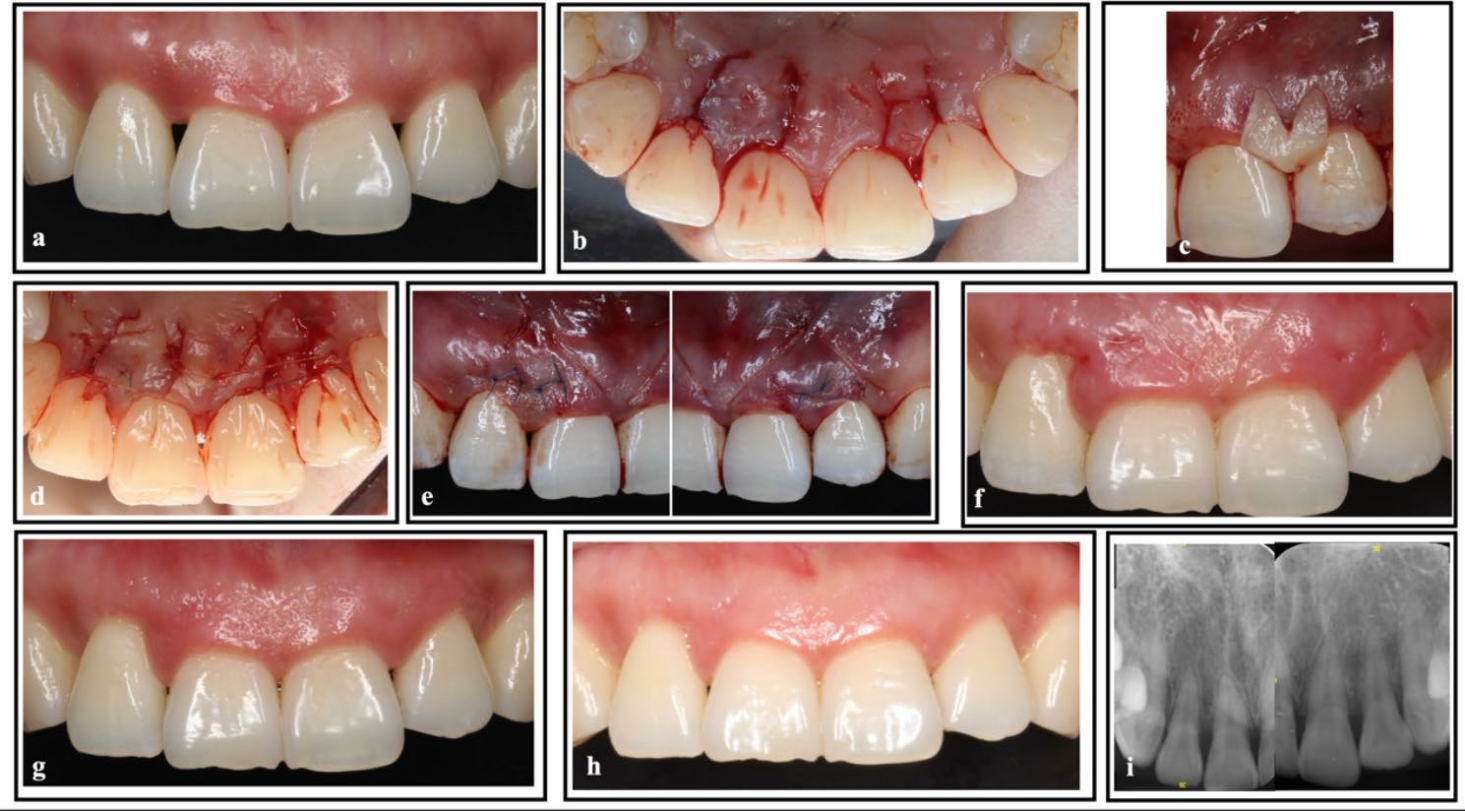
Surgical procedure and follow-up of Case 1. **(a)** Preoperative view showing the defects in the papilla between the right central and lateral incisors and between the left central and lateral incisors. **(b)** Vertical incision and cutback on the palatal side. **(c)** A CTG with a special shape. **(d) **Postoperative view after suturing at the palatal side. **(e)** Postoperative view after suturing at the labial side. **(f)** Suture removal 2 weeks after surgery. **(g)** At 3 months post-MIPT surgery, the height of the gingival papilla was restored. **(h)** At 12 months post-MIPT surgery, the gingival papilla in the surgery area was almost filled. **(i)** Radiographic image at baseline CTG, connective tissue graft; MIPT, modified interproximal tunneling

The first patient was a 22-year-old woman. Her maxillary anterior teeth had a compound crown form [18], and the distance between the alveolar crest and the contact point exceeded 5 mm. As a result, the gingival papilla was unable to fill the interproximal space, leading to the presence of a black triangle between the anterior teeth. This condition was diagnosed as mucogingival deformities. She was treated with MIPT to reconstruct the maxillary papillae between the right central and lateral incisors and between the left central and lateral incisors. The procedure and outcomes are shown in Fig. 1.

**Fig. 2:**
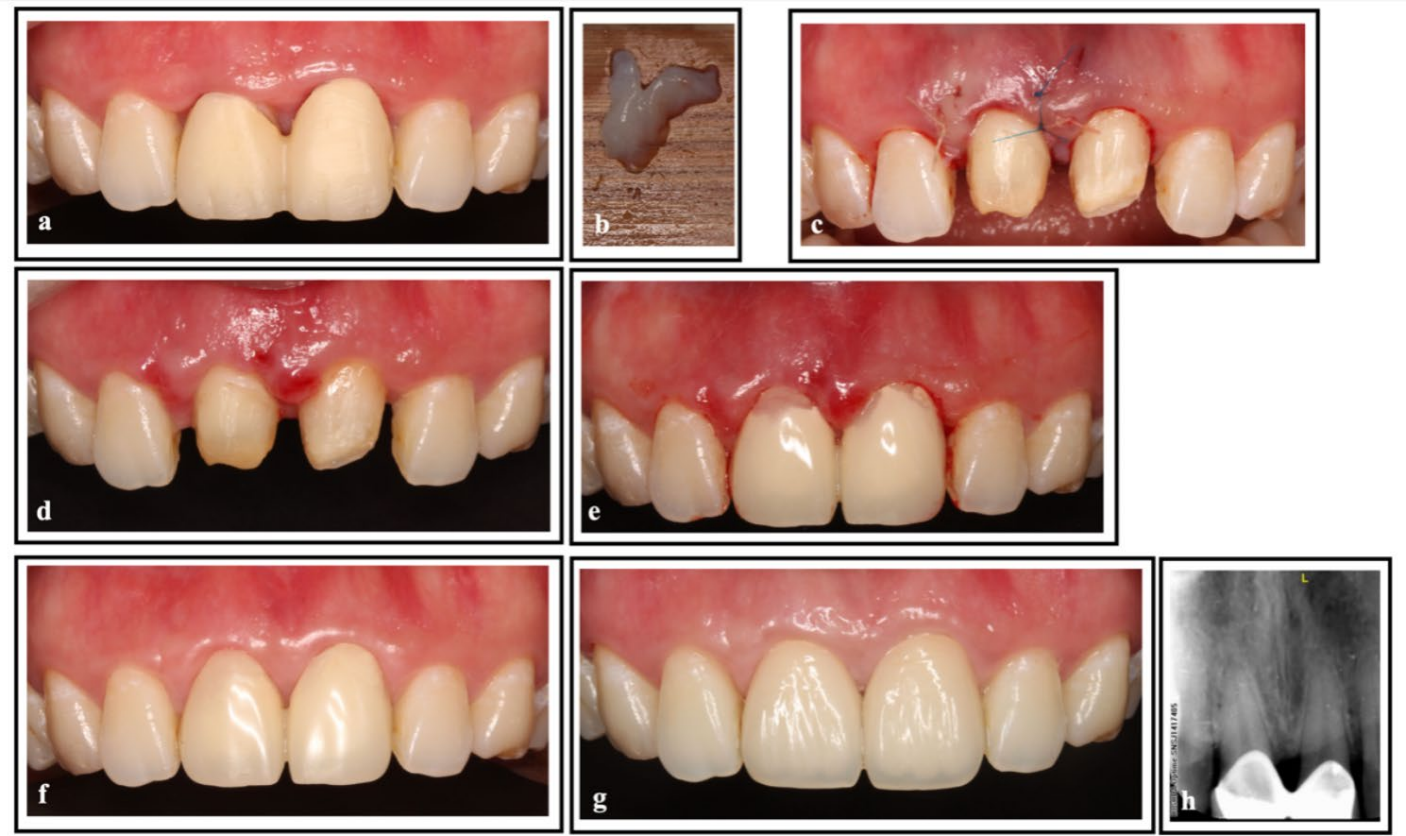
Surgical procedure and follow-up of Case 2. **(a)** Preoperative view showing the defects in the gingival papilla between the two central incisors and between the right maxillary central incisor and lateral incisor. **(b)** A CTG with a special shape. **(c)** The CTG was sutured in the defective areas of the gingival papillae. **(d)** Suture removal 2 weeks after surgery. **(e)** Provisional restoration was used 3 weeks after surgery. **(f)** A 3-month postoperative view of provisional crowns. **(g)** A 12-month postoperative view of permanent ceramic crowns showing that the surgery outcome was stable. **(h)** Radiographic image at baseline. CTG, connective tissue graft

The second patient was a 26-year-old woman. The restoration of two central incisors had to be removed due to repeated swelling of the gingiva of her maxillary central incisors. She was diagnosed with periodontitis and mucogingival deformities. After controlling the inflammation, a morphological defect in the gingival papillae was observed. To address the issue, MIPT was performed at the gingival papilla between the central incisors and between the maxillary right lateral and central incisors. Two provisional crowns were placed to shape the gingival papillae and were eventually replaced with permanent ceramic crowns when the soft tissue was stable. The procedure and outcomes are shown in Fig. 2.

**Fig. 3:**
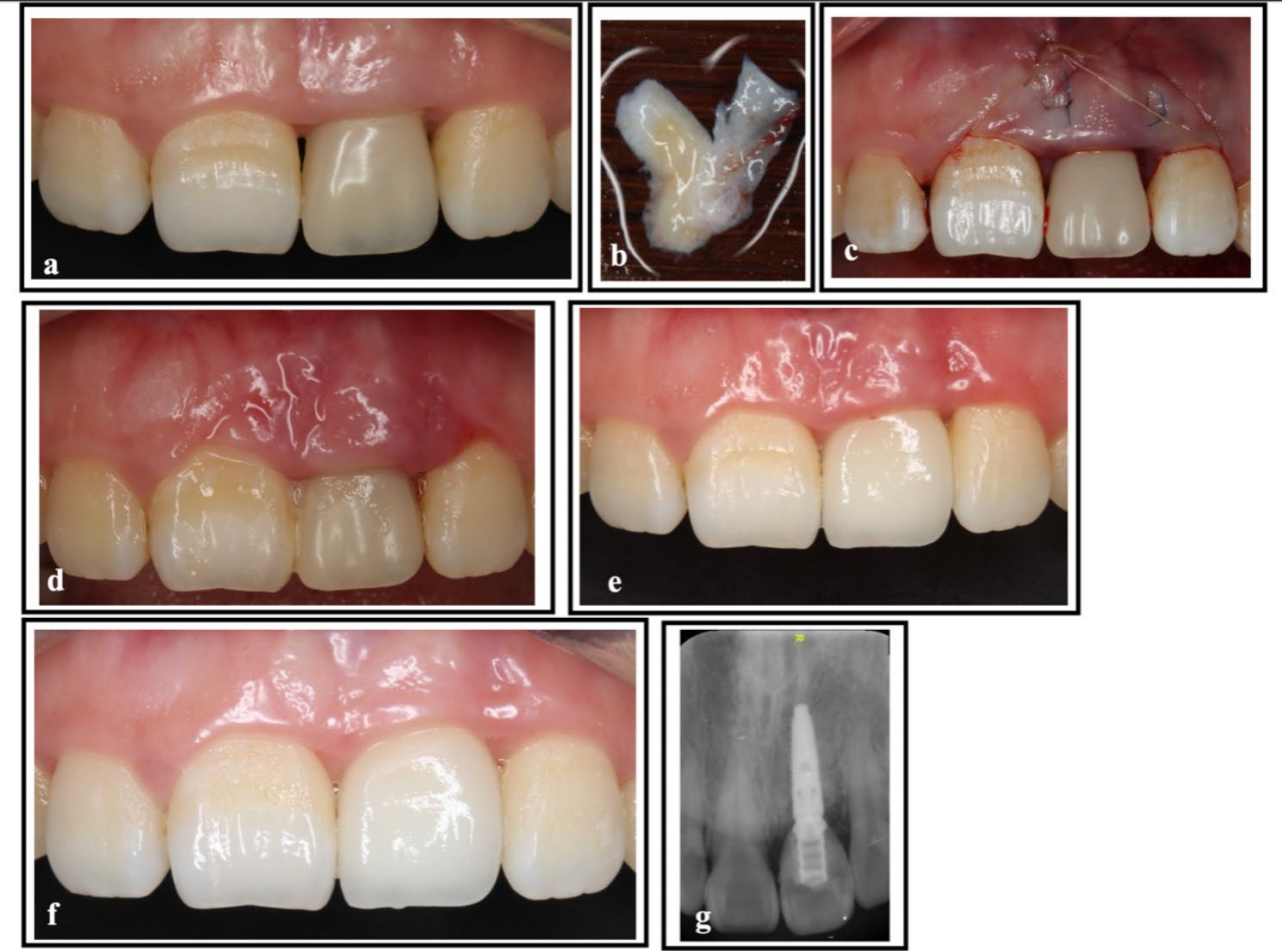
Surgical procedure and follow-up of Case 3. **(a)** Preoperative view showing papilla loss between central incisors and between the left maxillary central and lateral incisors. Note that the left maxillary central incisor is an implant. **(b)** Preoperative view of the CTG. **(c)** Postoperative view after suturing. **(d)** Suture removal 2 weeks after surgery. **(e)** A 3-month postoperative view of permanent ceramic crowns **(f) **At 12 months postsurgery, the papillae were completely recovered. **(g)** Radiographic image at baseline. CTG, connective tissue graft

The third patient, a 24-year-old woman, underwent implant restoration following the extraction of her left maxillary central incisor due to trauma. Despite six months of provisional crown restoration, the mesial and distal papillae still failed to fill the interproximal space. She was diagnosed with a dentition defect and mucogingival deformities. To address this issue, MIPT was performed, and the permanent crowns were restored once the papillae had completely recovered. The procedure and outcomes are shown in Fig. 3.

**Fig. 4:**
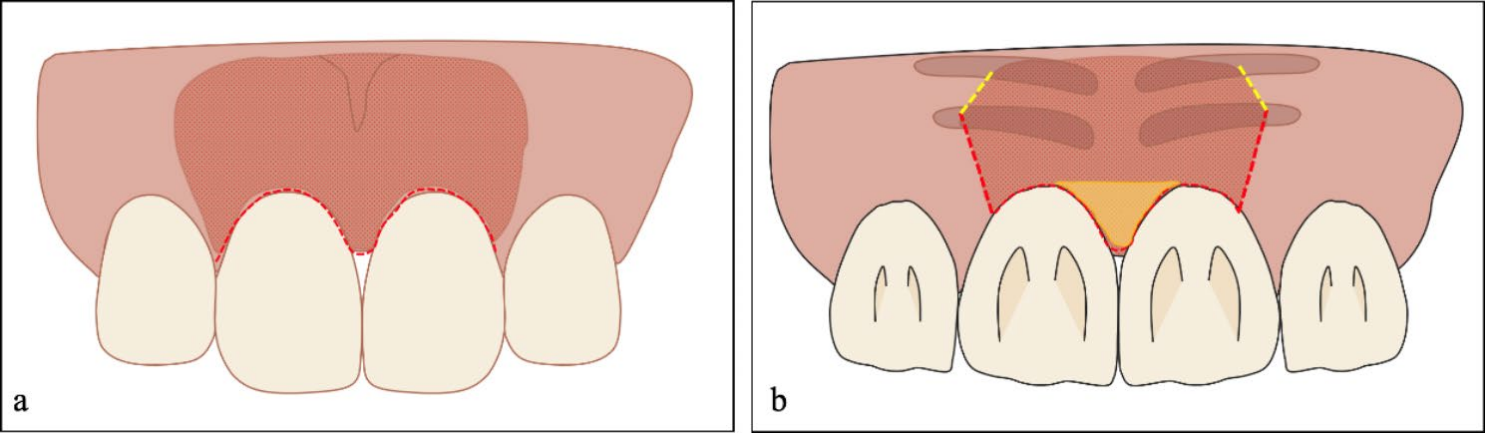
Schematic diagram of the surgical incisions on **(A)** the labial side and **(B)** the palatal side. The red dotted line indicates the position of the incisions. The yellow dotted line indicates cutback incisions. The yellow shaded area represents the full-thickness flap, while the red shaded area represents the split-thickness flap

All three patients were treated with MIPT to repair their gingival papilla defects. The preoperative and postoperative clinical examination results are shown in Tables 2 and 3. The operation was conducted according to the surgical technique proposed by Feuillet et al. [13] with some modifications. The surgeon was Jiachen Dong, and the anesthetist was Yue Liao. First, after local anesthetic infiltration (Articaine: epinephrine, 1:100,000), the deficient gingival papilla area was released and raised using two palatal vertical incisions and labial tunneling. Following local anesthesia, a crevicular incision was made along the labial and palatal sides of the entire papilla and the two adjacent teeth using a minimally invasive blade (#15c, Stoma, Germany). The incisive canal is reserved in the area of split-thickness flap, the periosteum around the incisive canal was preserved properly, which could provide sufficient blood supply. Second, labial mucoperiosteal tunneling was performed using a tunnel knife (Stoma, Germany), extending the range of gingival flap separation from the mesial to the distal, beyond the mucosal gingival junction. On the palatal side, two vertical 8–10-mm incisions with cutback incisions were made, and a split-thickness dissection was performed through the incisions. After complete relaxation, the entire papilla was raised from the alveolar ridge, creating space for positioning the CTG under the papilla (Fig. 4).

**Table 3:** MIPT results for the three patients included in the study

Subject	Interproximal papillae sites	Jemt papilla index score		Distance from the tip of the papilla to the contact point of the adjacent teeth (mm)	
		Preoperative	Postoperative	Preoperative	Postoperative
Case 1	#11, #12	1	2	3	0.5
	#21, #22	1	3	2	0
Case 2	#11, #21	2	3	1	0
	#11, #12	2	3	1	0
Case 3	#11, #21	2	3	3	0
	#21, #22	2	3	2	0

MIPT, modified interproximal tunneling

**Fig. 5:**
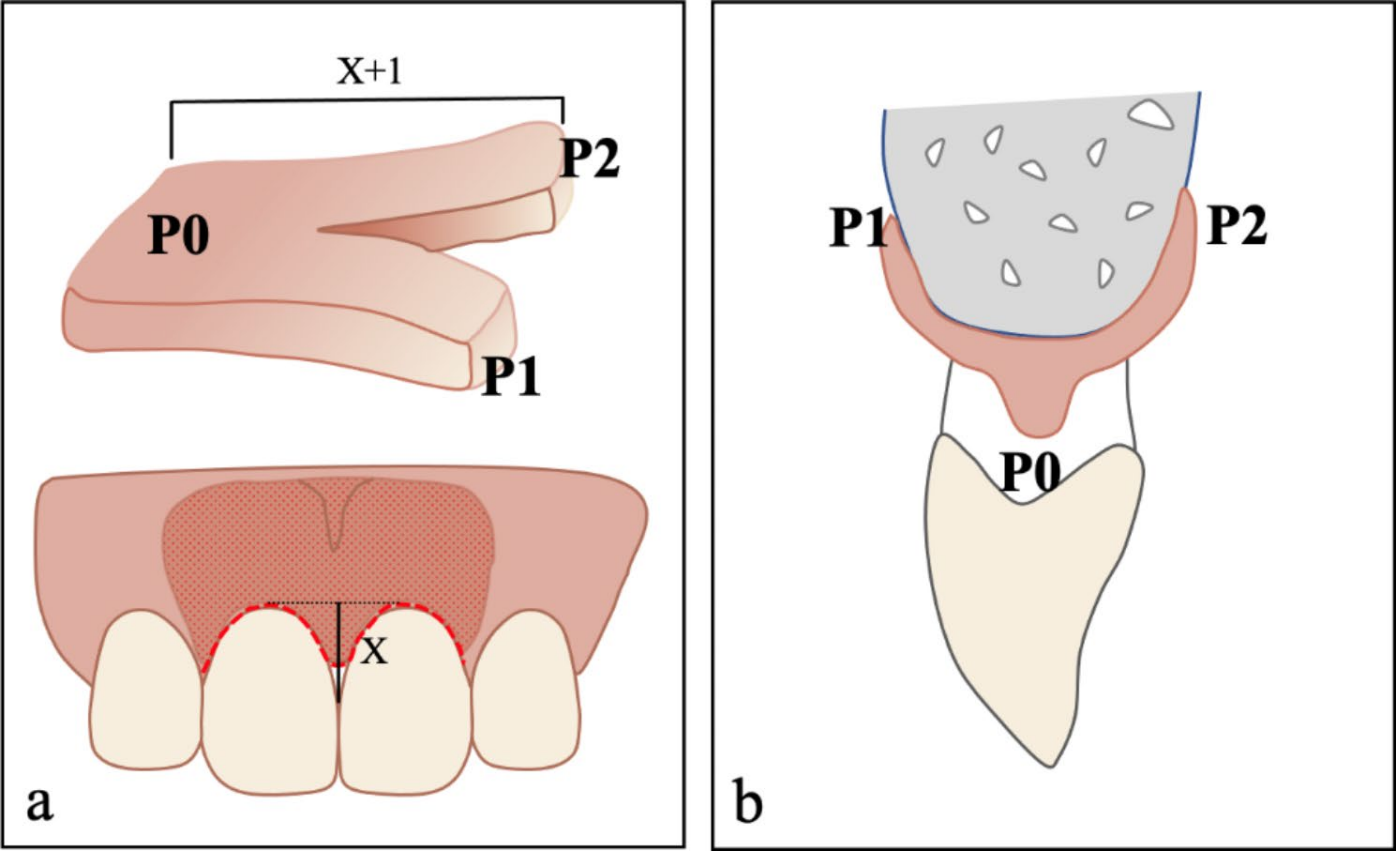
Schematic diagram of the shape and placement of the CTG. **(A)** Preparation of the CTG. **(B)** Placement of the CTG under the papilla. CTG, connective tissue graft

Then, the donor site was prepared. A sub-epithelial CTG with thickness of 2 mm was placed on the palatal area between the canine and second molar. A single horizontal incision, approximately 2 mm apical to the gingival margin of the maxillary teeth, was made to separate the epithelial layer [19]. A partial-thickness flap was made within the single incision using the #15c blade (Stoma, Germany), separating the sub-epithelial CTG from the connective tissue layer. Subsequently, a deeper horizontal incision was made at the same position as the original, reaching the upper layer of the periosteum and separating the connective tissue and periosteum. The sub-epithelial CTG of the mesial, distal, and root sides was then dissected internally. The graft length was 1 mm longer than the distance between the contact point and the base of the gingival papilla. The obtained CTG was separated from the center of the width and placed under the papilla through a vertical incision on the palatal side (Fig. 5). The suture (Polyglaction #5 − 0, Ethicon, USA) penetrated from the labial gingival flap, and then threaded through the gingival sulcus and CTG. Subsequently, the suture passed though gingival sulcus and threaded out of the labial gingival flap again. The CTG was tightly sutured with two lasso sutures on the labial side. Then, the same procedure was performed on the palatal side. The sutures were removed two weeks after the surgery. Ibuprofen 400 mg was used to manage postoperative pain. The patients were instructed to perform chemical plaque control using a 0.12% chlorhexidine rinse twice daily for 21 d following surgery. Recall visits were scheduled every three months postoperatively. Clinical parameters, including the Jemt papilla index [5] and the distance from the papilla tip to the interproximal contact point, were examined using a periodontal probe (UNC15, Hu-friedy, USA) at baseline and 12 months after the surgery.

Twelve months after the surgery, the height of the gingival papilla in the three cases was significantly higher than before surgery, with almost complete filling of the interproximal papillae. Except for one patient who missed their visit, the other two patients were satisfied with the aesthetic outcome and reported improved quality of life following the surgery. None of them suffered black triangle issues after the surgery. This study presents a modified technique to improve the predictability of papilla regeneration procedures, resulting in an average increase in the Jemt papilla score from 1.6 to 2.8 and an average decrease in the distance from the tip of the papilla to the contact point of the adjacent teeth from 2 mm to 0.08 mm (Table 1). MIPT can be successfully used for papillae regeneration between tooth/tooth, pontic/pontic, tooth/pontic, and tooth/implant.

## Discussion

Many studies have examined the reconstruction of the gingival papilla. Beagle [10] made a horizontal incision and two vertical incisions on the palatal side, separated the split-thickness flap, and sutured and attached the gingival flap to the buccal side. Han and Takei [11] pioneered the restoration of the missing gingival papilla through a combination of a semilunar incision and a CTG and obtained favorable surgical results. Papilla reconstruction has been applied to implant-supported restorations during second-stage surgery [20, 21].

In recent years, injectable hyaluronic acid has been used as a nonsurgical option for interdental papilla reconstruction [6, 9, 22]. However, the mechanism by which hyaluronic acid promotes tissue growth remains unclear. According to previous clinical case reports and clinical studies, achieving satisfactory results in reconstructing the gingival papilla is challenging due to several factors, such as limited papilla area, insufficient blood supply, surgical technique difficulties, and the lack of effective growth factors in nonsurgical treatments. Additionally, a recurrence of gingival papilla collapse has been observed in some nonsurgical treatments. Thus, surgery remains a preferred choice for treating gingival papilla defects.

IPT, proposed by Feuillet et al. [13], is a surgical method used to augment the gingival papilla. This method involves creating two palatal vertical incisions for tension reduction to lift the entire papilla. We have enhanced this approach by adding a cutback incision to the palatal vertical incision, allowing for increased relaxation. We called this method MIPT and presented three cases with favorable results. Based on our experience, successful gingival papilla reconstruction relies on the stability of the CTG, tension reduction in the gingival flap, and sufficient blood supply. Thus, the length of the two vertical incisions typically ranges from 8 to 10 mm, as most cases of gingival papilla defects require a height increase of approximately 2–3 mm. The palatal mucosa is extremely tight, making it difficult to reduce tension. A small incision length may impede the coronal restoration of the gingival papilla. Therefore, to enhance tension reduction and smoothly place the CTG in the appropriate position, we designed the vertical incisions to be 8–10 mm long, with an added cutback at the end.

A crucial step in this surgery is completely raising the gingiva. The base and labial sides of the gingival papilla are separated using the full-thickness technique, while the palatal side involves a split-thickness flap. Alongside a tunnel knife, a #13/14 subgingival curettage (Hu-Friedy) can be used, as its head can enter the base of the gingival papilla to facilitate separation from the bone. Completely raising the entire gingiva not only provides a recipient space under the papilla for CTG placement but also promotes the coronal advancement of the papilla.

In this study, three patients with gingival papilla defects in the maxillary anterior teeth underwent MIPT surgery. All the patients exhibited satisfactory results after 12 months. Based on our experience, reconstructing the gingival papilla around an implant is more difficult compared to a natural tooth, as periodontal ligaments provide a superior blood supply for the CTG. Thus, sufficient blood supply is even more critical for the papilla augmentation of implants. Therefore, ensuring the integrity of the gingival flap after separating the gingival papilla is crucial. In addition, the gingival biotypes could also affect the surgery effect. The probe will be visible in thin periodontal phenotypes (≤ 1 mm) and will not be visible in thick periodontal phenotypes (> 1 mm). Based on our study, all the patients were defined as thick periodontal phenotypes, which is more likely to achieve promising reconstruction effect compared with thin phenotypes. However, the effect of MIPT on thin periodontal phenotype is still unclear. Therefore, more patients with different periodontal phenotypes would be included in further studies for the comprehensive evaluation of MIPT.

## Conclusions

Reconstructing the gingival papilla is a challenging technique in periodontal mucogingival surgery. Here, we used MIPT to reconstruct the gingival papilla of natural teeth, an implant, and a prosthesis and obtained good results. Compared with the previous IPT technique, a cutback incision was added to the palatal vertical incision to enhance tension reduction and improve the CTG placement under the gingival papilla. The clinical implication of MIPT is to enhance the height and filling of the reconstructed gingival papilla within the interproximal space. To determine the definitive effects of MIPT, additional research with longer follow-up needs to be conducted.

## Data Availability

The datasets used and/or analyzed during the current study are available from corresponding author on reasonable request.
